# Forizymes – functionalised artificial forisomes as a platform for the production and immobilisation of single enzymes and multi-enzyme complexes

**DOI:** 10.1038/srep30839

**Published:** 2016-08-09

**Authors:** Franziska Visser, Boje Müller, Judith Rose, Dirk Prüfer, Gundula A. Noll

**Affiliations:** 1 University of Münster, Institute of Plant Biology and Biotechnology, Münster, 48143, Germany; 2 Fraunhofer Institute for Molecular Biology and Applied Ecology IME, Münster, 48143, Germany

## Abstract

The immobilisation of enzymes plays an important role in many applications, including biosensors that require enzyme activity, stability and recyclability in order to function efficiently. Here we show that forisomes (plant-derived mechanoproteins) can be functionalised with enzymes by translational fusion, leading to the assembly of structures designated as forizymes. When forizymes are expressed in the yeast *Saccharomyces cerevisiae*, the enzymes are immobilised by the self-assembly of forisome subunits to form well-structured protein bodies. We used glucose-6-phosphate dehydrogenase (G6PDH) and hexokinase 2 (HXK2) as model enzymes for the one-step production and purification of catalytically active forizymes. These structures retain the typical stimulus-response reaction of the forisome and the enzyme remains active even after multiple assay cycles, which we demonstrated using G6PDH forizymes as an example. We also achieved the co-incorporation of both HXK2 and G6PDH in a single forizyme, facilitating a two-step reaction cascade that was 30% faster than the coupled reaction using the corresponding enzymes on different forizymes or in solution. Our novel forizyme immobilisation technique therefore not only combines the sensory properties of forisome proteins with the catalytic properties of enzymes but also allows the development of multi-enzyme complexes for incorporation into technical devices.

Enzymes are used as catalysts in a wide range of biotechnology-based processes. They achieve high selectivity, work under mild conditions and are environmentally friendly. However, enzymes often lack the necessary stability under process conditions, thus limiting their applications^1^. This issue can be overcome using immobilised enzymes, which are not only more stable than their soluble counterparts, they can also be used continuously, recycled efficiently and separated easily from the product, enabling cost-effective biocatalytic processes^2^. Standard immobilisation techniques include binding to carriers (by adsorption, ionic attraction or covalent bonds), entrapment/encapsulation, and chemical cross-linking^3^. Because the choice of method influences the properties of the immobilised enzyme, each application must be considered on a case-by-case basis^2^. The physical and chemical properties of carriers not only affect the final catalytic properties of the enzyme in terms of activity, stability and selectivity, they also determine which application fields are suitable^4^. This has created a demand for novel and innovative carriers that fulfil the requirements of specific applications^5^. The most promising candidates are often ‘smart polymers’ that undergo conformational changes triggered by certain stimuli, such as a change in pH, temperature or salt concentration^2^. For example, enzymes have been immobilised onto the thermoresponsive polymer poly-*N*-isopropylacrylamide (polyNIPAM) using conventional immobilisation strategies^6^,^7^,^8^. However, both carrier production and the immobilisation process have a significant environmental impact, increasing the need for more sustainable carriers and mild immobilisation techniques^9^.

Self-assembling protein systems fulfil the criteria listed above, hence S-layer proteins, silaffin and peptide hydrogels are examples of protein-based systems that have already been used as carriers following enzyme immobilisation by translational fusion^10^,^11^,^12^. Furthermore, protein assemblies comprising cellulose binding domains, cohesin and dockerin units or heterotrimeric proliferating cell nuclear antigen have been used as scaffolds for the co-immobilisation of multiple enzymes, thus mimicking naturally occurring multi-enzyme reaction cascades^13^,^14^,^15^. The spatial organisation of such systems brings the co-immobilised enzymes into close proximity, allowing the direct transfer of reaction intermediates between enzyme active sites, a process described as substrate channelling^16^. This reduces the diffusion of intermediates into the bulk phase and can substantially increase reaction rates, thus improving reaction efficiency and offering a convenient platform for biotechnology-based applications. To the best of our knowledge, a self-assembling protein system that can immobilise multiple enzymes while retaining stimulus-response characteristics has not yet been considered for enzyme immobilisation.

We therefore investigated the potential of plant-derived mechanoproteins (forisomes) as a platform for the immobilisation of enzymes. Forisomes are self-assembled multiprotein complexes 1–5 μm in diameter and 10–40 μm in length that are present in the phloem of fabaceaen plants. When the plant is wounded, forisomes undergo a Ca^2+^-induced, ATP-independent and reversible conformational change from a condensed into a dispersed state to prevent the loss of photoassimilates by plugging the injured sieve element^17^,^18^. Electrically-induced pH shifts also trigger forisomes to undergo conformational changes *in vitro*^19^, suggesting they could be developed as smart biomaterials for applications such as biosensors, micro-grippers or valves in microfluidic devices^20^. The latter was demonstrated by placing a forisome in a microchannel and adding Ca^2+^, which caused the expanding forisome to block the flow in the channel in a manner reminiscent of the natural function of forisomes^21^. Initially such devices were based on forisomes laboriously isolated from phloem tissue^21^,^22^. More recently, four genes encoding forisome subunits were identified in *Medicago truncatula* and were assigned to the *sieve element occlusion by forisome* (*SEO-F*) gene family^23^. The heterologous expression of two subunits (MtSEO-F1 and MtSEO-F4) allows the bulk production of artificial forisomes suitable for downstream technical applications^24^. These subunits appear to be primarily responsible for the formation of the native forisome body, whereas MtSEO-F2 appears to fine-tune the geometric proportions of forisomes and hence their activity^25^. The function of MtSEO-F3 remains unclear^26^. Artificial forisomes produced by expressing MtSEO-F1 or MtSEO-F4 in the yeast *Saccharomyces cerevisiae* also self-assemble into micro-scale forisome structures even when small additional tags are translationally fused to the forisome subunits^24^,^27^. However, the potential to display more complex tags such as enzymes on artificial forisomes has not been investigated.

Here, we used fluorescent reporter proteins to investigate the optimal combination of MtSEO-F1/MtSEO-F4 subunits and the most appropriate fusion site (N-terminus or C-terminus) for the production in *S. cerevisiae* of robust, high-quality forizymes, i.e. forisomes functionalised with catalytic properties by the assembly of subunits comprising forisome–enzyme fusion proteins. We then produced catalytically active forizymes containing immobilised glucose-6-phosphate dehydrogenase (G6PDH) or hexokinase 2 (HXK2). The G6PDH forizymes retained their enzymatic activity even after multiple assay cycles, confirming that forizymes are durable and suitable for repetitive use. Two-step enzymatic reactions were then carried out using dual-functionalised forizymes displaying both HXK2 and G6PDH. We conclude that forizymes are promising and versatile multi-protein complexes that are suitable for applications requiring immobilised enzyme cascades, including biotransformation reactions and biosensors in the fields of analytical chemistry and medicine.

## Results and Discussion

### Identification of appropriate MtSEO-F subunits for translational fusion with non-related proteins

**Figure 1:**
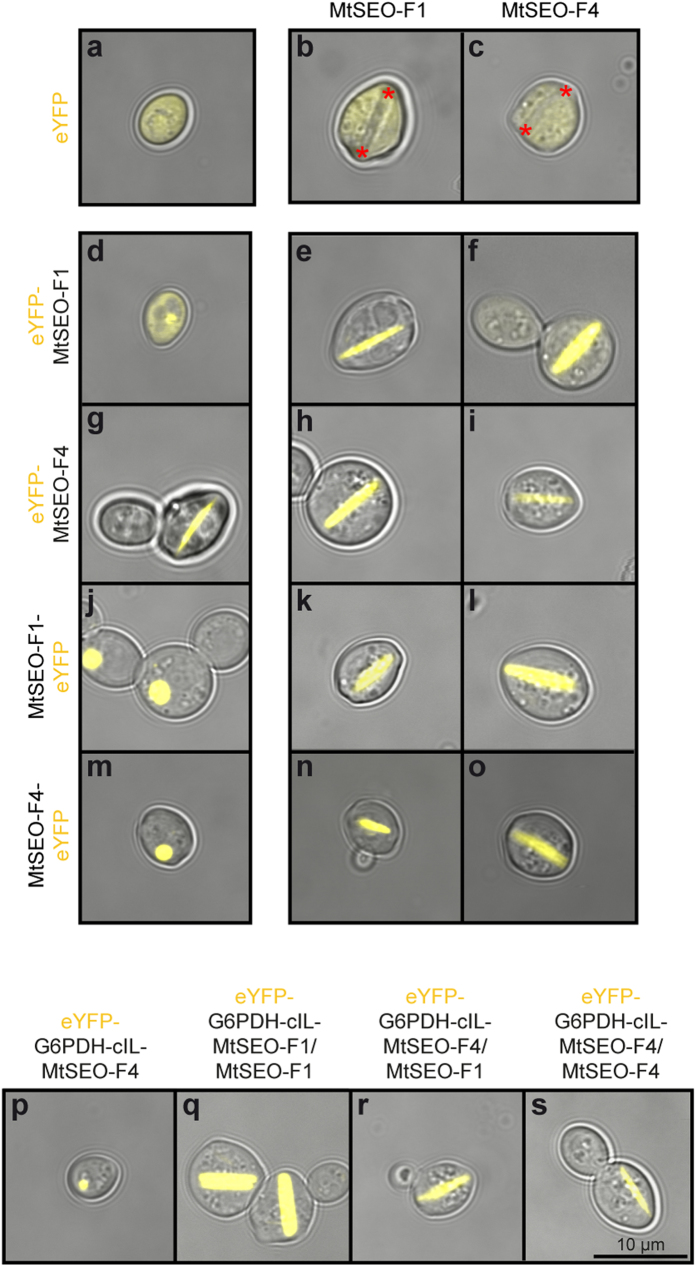
Production of eYFP-tagged artificial forisomes in *Saccharomyces cerevisiae*. Representative confocal microscopy images of control cells expressing eYFP alone (**a**) or in combination with the untagged MtSEO-F1 (**b**) or MtSEO-F4 (**c**) subunits show cytosolic fluorescence as well as non-fluorescent artificial forisomes (indicated by red asterisks) in (**b**,**c**). Yeast cells expressing N-terminal eYFP-MtSEO-F fusion proteins (**d**–**i**) or C-terminal MtSEO-F-eYFP fusion proteins (**j**–**o**) either alone or in combination with each untagged MtSEO-F protein reveal the most suitable combinations for functionalisation by forming fluorescent forisome structures. (**p**–**s**) Yeast cells expressing candidate MtSEO-F combinations as eYFP-glucose-6-phosphate dehydrogenase (G6PDH)-cIL-MtSEO-F fusion proteins for the confirmation of functional forisome assembly when larger enzyme tags are present.

MtSEO-F1 and MtSEO-F4 subunits can assemble into artificial forisomes in *S. cerevisiae* and can be functionalised by the translational fusion of small tags. This offers promising applications for the development of technical devices^20^,^24^,^27^. In order to functionalise artificial forisomes with larger enzyme tags, we first identified the most appropriate combination of MtSEO-F1 and MtSEO-F4 subunits as well as the best translational fusion site. This was achieved by fusing each subunit to the enhanced yellow fluorescent protein (eYFP) for easy visualisation, and analysing the robustness of the assembly of combinations of N/C-terminal eYFP-tagged and untagged MtSEO-F1 and MtSEO-F4 constructs in yeast cells. As controls, eYFP was expressed either alone (Fig. 1a) or in combination with the untagged MtSEO-F1 (Fig. 1b) or MtSEO-F4 (Fig. 1c). All three controls resulted in diffuse cytosolic yellow fluorescence. Untagged MtSEO-F1 and MtSEO-F4 artificial forisomes were also visible because each subunit can spontaneously assemble into forisomes, the ends of which are indicated by red asterisks in Fig. 1b,c. Each subunit was expressed as either an N-terminal (Fig. 1d,g) or C-terminal (Fig. 1j,m) eYFP fusion, and we found that artificial forisomes assembled only in the case of the N-terminal eYFP-MtSEO-F4 fusion construct (Fig. 1g). This configuration was therefore deemed the only suitable approach for the production of homomeric functionalised artificial forisomes. In contrast, fluorescent heteromeric forisome bodies were visible in cells co-transformed with all tagged/untagged combinations of the subunits, i.e. eYFP-MtSEO-F1/MtSEO-F1 (Fig. 1e), eYFP-MtSEO-F1/MtSEO-F4 (Fig. 1f), eYFP-MtSEO-F4/MtSEO-F1 (Fig. 1h), eYFP-MtSEO-F4/MtSEO-F4 (Fig. 1i), MtSEO-F1-eYFP/MtSEO-F1 (Fig. 1k), MtSEO-F1-eYFP/MtSEO-F4 (Fig. 1l), MtSEO-F4-eYFP/MtSEO-F1 (Fig. 1n) and MtSEO-F4-eYFP/MtSEO-F4 (Fig. 1o). Initially, all MtSEO-F1/MtSEO-F4 subunit combinations therefore seemed equally suitable for functionalisation, reflecting the relatively minor differences in their ability to assemble. This resembles their role in nature, i.e. the primary function of both proteins appears to be the establishment or stabilisation of forisome structures^25^,^28^. However, in addition to cells exclusively containing artificial forisomes, we also observed cells with fluorescent inclusion bodies or diffuse cytosolic fluorescence. Therefore, the number of cells containing artificial forisomes, inclusion bodies or diffuse cytosolic fluorescence was determined by microscopy from a pool of three randomly selected yeast colonies in order to identify the optimal combination of tagged MtSEO-F subunits leading to the most robust assemblies, i.e. those producing artificial forisomes in the greatest proportion of cells (Table 1a). Artificial forisomes were predominant in the yeast cells transformed with the N-terminal fusion constructs eYFP-MtSEO-F4 (alone), eYFP-MtSEO-F1/MtSEO-F1, eYFP-MtSEO-F4/MtSEO-F1 and eYFP-MtSEO-F4/MtSEO-F4 (Table 1a), whereas C-terminal fusion constructs showed a greater tendency to form by-products such as inclusion bodies, which could be caused by a disturbed assembly process, the single exception being MtSEO-F4-eYFP/MtSEO-F4. Translational fusions can influence protein folding, and functional differences between N-terminal and C-terminal fusions of the same protein components have been reported previously^29^. This suggests that C-terminal translational fusions inhibit interactions between or the assembly of MtSEO-F proteins and thus the overall forisome structure^27^. Because the N-terminal fusion strategy was generally more successful, all the C-terminal fusion constructs were excluded from further analysis.

Next, we investigated the ability of the productive MtSEO-F combinations identified above to display larger enzyme fusion proteins. We chose G6PDH for initial tests because the enzyme activity can be measured using a simple assay. G6PDH catalyses the oxidation of D-glucose-6-phosphate to 6-phospho-D-gluconate while reducing NADP^+^ to NADPH, which can be monitored by spectrophotometry. We prepared eYFP-G6PDH-MtSEO-F translational fusion protein constructs so that the correct assembly of the forisome subunits could be visually confirmed. The constructs also included the synthetic linker cIL (comprising the Myc epitope, an immunoglobulin A1 protease cleavage site and a flexible linker region) between the C-terminus of G6PDH and the N-terminus of the MtSEO-F subunit (see methods section), allowing antibody detection and/or proteolytic cleavage of the fusion protein. The resulting constructs were named eYFP-G6PDH-cIL-MtSEO-F4, eYFP-G6PDH-cIL-MtSEO-F1/MtSEO-F1, eYFP-G6PDH-cIL-MtSEO-F4/MtSEO-F1 and eYFP-G6PDH-cIL-MtSEO-F4/MtSEO-F4. All four constructs were expressed in yeast to determine which assemblies were most robust. As shown in Fig. 1p–s, the ability of the forisome subunits to assemble was retained in the heteromeric combinations of tagged/untagged pairs (Fig. 1q–s) but the homomeric eYFP-G6PDH-cIL-MtSEO-F4 construct was no longer able to form protein assemblies (Fig. 1p). This suggests that the larger fusion tag interferes with assembly when it is present on all subunits, perhaps by steric hindrance or the specific abolition of necessary binding interactions, whereas the presence of intact subunits without fusion proteins allows such interactions to be preserved in heteromeric assemblies. A similar effect has been reported for the self-assembly of functionalised viral core proteins into capsid-like particles used in vaccine display systems^30^. When core proteins are fused with heterologous proteins, they are too bulky to assemble on their own, but co-assembly with wild-type core proteins rescues particle formation presumably by preventing steric hindrance caused by adjacent subunits carrying fusion tags.

Construct eYFP-G6PDH-cIL-MtSEO-F4 (Fig. 1p) was therefore excluded from further studies. The combination eYFP-G6PDH-MtSEO-cIL-F1/MtSEO-F1 was the most robust, assembling into artificial forisomes in 61% of the fluorescent cells, followed by eYFP-G6PDH-cIL-MtSEO-F4/MtSEO-F4 (49%) and eYFP-G6PDH-cIL-MtSEO-F4/MtSEO-F1 (42%). The robustness of the constructs is summarised in Table 1b.

### Selection of the most productive MtSEO-F subunit combination for G6PDH forizymes

We next investigated the catalytic activity of the three most robust combinations identified above as a second criterion for the selection of the optimal MtSEO-F subunit combination. Therefore, new constructs were prepared lacking the eYFP component of the fusion protein to minimise any interference with the enzymatic activity of G6PDH. The resulting construct combinations were named G6PDH-cIL-MtSEO-F1/MtSEO-F1, G6PDH-cIL-MtSEO-F4/MtSEO-F1 and G6PDH-cIL-MtSEO-F4/MtSEO-F4.

Yeast cells expressing these three constructs were analysed for G6PDH activity by monitoring the increase in absorbance at 340 nm due to the conversion of NADP^+^ to NADPH in the forisome fraction of disrupted cells. The activity of each of the three fractions was significantly higher than fractions of control cells expressing only MtSEO-F1 (p < 0.001, Kruskal-Wallis one-way analysis of variance (ANOVA) on ranks, post hoc Tukey test, n = 12, Supplementary Fig. S1) but there was no significant difference among the three combinations. However, the highest activity (30-fold higher activity than control cells) was achieved for individual colonies expressing the combination G6PDH-cIL-MtSEO-F1/MtSEO-F1 and this combination also produced the most robust artificial forisomes (Table 1b). Therefore, forisomes based on G6PDH-cIL-MtSEO-F1/MtSEO-F1 were used for subsequent studies.

### Production and molecular characterisation of forizymes

**Figure 2:**
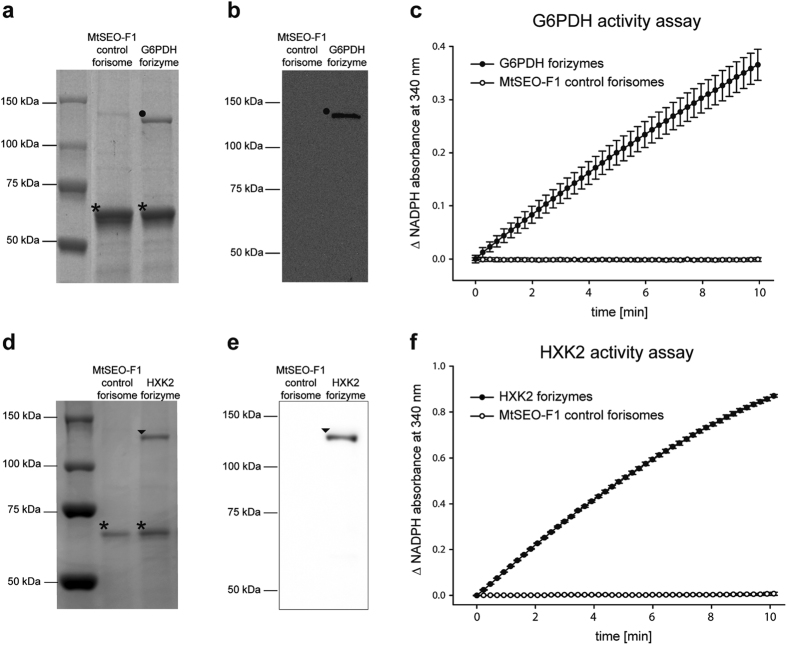
Production and catalytic activity of G6PDH and HXK2 forizymes. The forizymes were composed of G6PDH-cIL-MtSEO-F1 and MtSEO-F1 subunits (G6PDH forizymes) or HXK2-HAL-MtSEO-F1 and MtSEO-F1 subunits (HXK2 forizymes) and were produced in *S. cerevisiae* cells. (**a**) SDS-PAGE analysis for the verification of G6PDH forizyme production. Control forisomes composed solely of MtSEO-F1 were produced and purified simultaneously. Symbols: *untagged MtSEO-F1, •G6PDH-cIL-MtSEO-F1 fusion. (**b**) Western blot for the detection of the G6PDH-cIL-MtSEO-F1 fusion protein (•) in purified G6PDH forizymes using a Myc epitope-specific antibody (the Myc epitope is part of the synthetic linker cIL). The antibody did not detect the purified control forisomes composed solely of MtSEO-F1. (**c**) Activity assay for the purified G6PDH forizymes and MtSEO-F1 control forisomes based on measuring the formation of NADPH by monitoring the absorbance at 340 nm in a G6PDH enzyme assay. (**d**) SDS-PAGE analysis for the verification of HXK2 forizyme production. Control forisomes composed solely of MtSEO-F1 were produced and purified simultaneously. Symbols: *untagged MtSEO-F1, ▼HXK2-HAL-MtSEO-F1 fusion. (**e**) Western blot for the detection of the HXK2-HIL-MtSEO-F1 fusion protein (▼) in purified HXK2 forizymes using an HA-specific antibody. The antibody did not detect the purified control forisomes composed solely of MtSEO-F1. (**f**) Activity assay for the purified HXK2 forizymes and MtSEO-F1 control forisomes based on a coupled reaction with soluble G6PDH leading to the formation of NADPH. Each value was corrected for the blank measurement. Graphs show means ± SD of technical triplicates representing one of three independent forizyme purifications.

The G6PDH-cIL-MtSEO-F1/MtSEO-F1 forisomes, hereafter described as G6PDH forizymes, were purified by density gradient centrifugation along with artificial control forisomes solely comprising homomeric assemblies of untagged MtSEO-F1. We isolated 2.8 ± 1.1 × 10^7^ forizymes from 200 ml of yeast culture, representing a total yield of 34 ± 5 μg G6PDH forizyme protein. The purified forizymes were separated by SDS-PAGE (Fig. 2a), revealing two prominent protein bands corresponding to the molecular masses of the untagged MtSEO-F1 protein (74.8 kDa, indicated by asterisks) and the G6PDH-cIL-MtSEO-F1 fusion protein (135.7 kDa, indicated by a dot). Additionally, the 135.7 kDa G6PDH-cIL-MtSEO-F1 fusion protein was detected by western blot analysis using an antibody specific for the Myc epitope (cIL, Fig. 2b), thereby confirming that G6PDH-linked MtSEO-F1 subunits are present as full-length proteins in the purified forizymes. We subsequently investigated the catalytic activity of the purified G6PDH forizymes by monitoring the production of NADPH via the absorbance at 340 nm (Fig. 2c). The initial rate of substrate turnover was used to calculate a specific activity of 10,800 ± 3,300 U/g G6PDH forizyme protein. It is remarkable that the G6PDH molecules retain catalytic activity within the forizyme assembly because active G6PDH is a homodimer^31^. The assembly of the MtSEO-F1 subunits must therefore allow the fused G6PDH monomers to form active homodimers. However, we cannot exclude the possibility that some G6PDH molecules remain in the form of inactive monomers and that the forizymes have not yet achieved their maximum potential activity.

Once the immobilisation of G6PDH on forizymes was demonstrated as a proof of concept, the next step was to produce forizymes with a different model enzyme to confirm its potential as a platform technology. We chose HXK2 because it has the ability to act in a cascade with G6PDH. HXK2-functionalised forizymes were developed using the same strategy described above for G6PDH forizymes, but the Myc epitope in the construct was replaced with a hemagglutinin (HA) tag so that each protein could be detected separately. The synthetic linker of this construct was designated as HAL and the resulting HXK2-HAL-MtSEO-F1/MtSEO-F1 forisomes are described as HXK2 forizymes hereafter. SDS-PAGE analysis (Fig. 2d) confirmed the presence of both components of the HXK2 forizyme, namely the HXK2-HAL-MtSEO-F1 subunits (131.7 kDa, indicated by an arrowhead) and the MtSEO-F1 subunits (74.8 kDa, indicated by asterisks). The full length HXK2-HAL-MtSEO-F1 fusion protein was detected by western blot using HA-specific antibodies to confirm the presence of the full-length fusion protein in the forizyme (Fig. 2e). Next, the catalytic activity of HXK2 forizymes was determined in an enzyme assay (Fig. 2f), revealing a specific HXK2 activity of 25,700 ± 2,900 U/g forizyme. The specific activity in terms of single enzyme units in forizymes can be calculated by dividing the forizyme-specific activity by the catalytic mass of each enzyme (Table 2). Thereby we calculate HXK2 activity to be approximately 525,000 U/g enzyme unit and G6PDH activity to be approximately 154,000 U/g enzyme unit in the context of the forizyme. The HXK2 activity matches the literature values for pure HXK2 (500,000–600,000 U/g)^32^ indicating that immobilisation has little impact on enzyme activity, whereas the G6PDH activity is lower than that described for pure G6PDH (350,000 U/g)^33^ indicating that immobilisation has a greater effect on this enzyme.

To the best of our knowledge, the activities of the two forizymes are the highest activities ever reported for immobilised G6PDH and HXK produced in *S. cerevisiae*. Previously, G6PDH was reported to have an activity of 176 U/g when immobilised on polyacrylamide beads^34^ and 1,000–1,750 U/g when immobilised on agarose beads (G5506 Sigma-Aldrich). Similarly, HXK was reported to have an activity of 4,700 U/g when immobilised on nylon^35^ and 1,200–2,000 U/g when immobilised on agarose beads (H2005 Sigma-Aldrich). Unlike synthetic beads, the enzymes in forizymes are produced and immobilised in a single step, which is highly advantageous because there is no need to purify the enzyme before immobilisation, as required in conventional strategies^2^. The forizymes can be purified by centrifugation or filtration, which offers a simple and cost-effective procedure for large-scale production^24^. Other immobilisation methods and protein-based self-assembly systems for enzyme immobilisation require further production steps such as preliminary activation of the carrier, cross-linking procedures involving hazardous chemicals or the initiation of the assembly process^3^,^10^,^12^,^36^. This is not necessary with forizymes because the translational fusion facilitates the direct incorporation of the enzyme into the forisome matrix by exploiting spontaneous forisome self-assembly without an external stimulus. Remarkably, the fused G6PDH and HXK2 enzymes remain catalytically active and the translational fusion prevents the release of enzymes from the forisome carrier, which is important because enzyme leaching is a major drawback of many immobilisation approaches^2^. Forizymes therefore reduce the risk of enzyme contamination in the product stream in addition to offering a sustainable and biodegradable resource^20^.

### Characterisation of forizyme composition and performance

To gain further insight into the characteristics of forizymes as an enzyme immobilisation platform, the protein composition and catalytic efficiency of G6PDH and HXK2 forizymes were analysed based on subunit quantification by SDS-PAGE and enzyme activity assays. The quantitative comparison of subunit types suggested that in both types of forizyme the enzyme-MtSEO-F1 fusion protein comprises 13–19% of the forizyme by mass and the untagged MtSEO-F1 subunit represents 81–87% of the mass, assuming that all forizymes assemble in broadly the same manner and are proportionally similar in their composition (Table 2a). The marginal differences in composition between the two types of forizyme suggest that a tagged-to-untagged subunit ratio of 1:15–20 is likely to be representative of forizymes prepared using this strategy. Therefore, the catalytic mass of the forizyme is 5–7%, i.e. the proportion made up of the enzyme, whereas the non-catalytic mass is 93–95%, i.e. the proportion made up of the untagged subunit and the MtSEO-F1 component of the fusion protein. This is a good value for carrier-bound enzymes because the non-catalytic mass of other insoluble carrier materials is usually in the range of 90% to >99%^2^. Furthermore, the loading capacity of forizymes could be increased in the future by reducing the amount of catalytically inactive biomass, e.g. by using deletion variants of the MtSEO-F1 protein.

**Figure 3:**
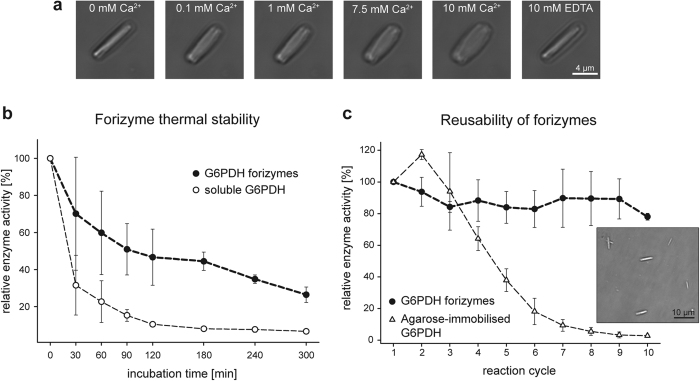
Stimulus-response characteristics and stability of forizymes. (**a**) Microscopic images of G6PDH forizyme conformational changes caused by the addition of Ca^2+^ and subsequent removal. (**b**) Thermal stability of forizymes and soluble G6PDH. Both enzyme preparations were incubated at 40 °C and aliquots were withdrawn at different time points to determine residual enzyme activity. (**c**) To demonstrate recyclability, forizyme and agarose-immobilised G6PDH activity was monitored during multiple reaction cycles. Forizymes were centrifuged to the bottom of the reaction well prior to the reaction (small picture). Measurements are shown as means ± SD of three independent experiments for each purification. Relative enzyme activity is shown as a percentage of initial activity.

Forisomes respond to Ca^2+^ by undergoing a reversible, force-generating conformational change, making them potentially attractive as smart biomaterials in microfluidic devices, lab-on-a-chip systems and biosensors. We next sought to establish whether the forisome reaction is retained following the functionalisation of the MtSEO-F1 subunit with enzymes, so we exposed G6PDH forizymes as a representative case study to increasing Ca^2+^ concentrations (Fig. 3a). In the absence of Ca^2+^, the G6PDH forizymes formed a spindle-like structure, but this changed into the dispersed conformation following the application of 0.1, 1, 7.5 or 10 mM Ca^2+^. When the Ca^2+^ was removed by adding 10 mM EDTA, the G6PDH forizymes changed back into the original spindle-like structure, demonstrating the reversibility of the reaction. We measured the enzymatic activity of the G6PDH forizymes at the different Ca^2+^ concentrations and found that the changing conformation has no significant effect (p = 0.055, one-way ANOVA, Supplementary Fig. S2). Forizymes therefore appear to retain both the stimulus-response characteristics of the parental forisome and the catalytic activity of the enzymatic component.

Immobilised enzymes are often more stable than their soluble counterparts^37^. We therefore analysed G6PDH forizymes in terms of thermal stability compared to soluble G6PDH. Both enzyme preparations were incubated at 40 °C for 5 h and aliquots were taken at different time points to determine the residual activity (Fig. 3b). The activity of the G6PDH forizymes was reduced by 50% after incubation for 120 min, but remained above 30% of the initial activity for 240 min. In contrast, the activity of soluble G6PDH was reduced by ~70% after incubation for 30 min and by ~90% after 120 min. The results are comparable to previous studies using G6PDH immobilised on agarose or dextran carriers (Sepharose and Sephadex, respectively) in which the enzyme preparations retained 70–90% of their initial activity after 15 min incubation at 40 °C^38^,^39^. The forizyme platform therefore improves the thermal stability of the enzyme, probably because the dense forizyme assembly restricts the mobility of the enzyme and prevents the dissociation of the active heterodimer, thus stabilizing the quaternary structure^40^. We will consider whether forizymes are useful platforms for the immobilisation of more challenging (e.g. unstable, toxic or membrane-bound) enzymes in future studies.

A major advantage of immobilised enzymes is their reusability. Therefore, we next determined the activity of purified G6PDH forizymes over multiple reaction cycles, using agarose-immobilised G6PDH as a comparator (Fig. 3c). The forizymes were briefly centrifuged to settle them in the wells of a microtiter plate (Fig. 3c, small picture) thus exploiting their sedimentation propensity and adherence to the well surface^28^. The enzymatic activity of the G6PDH forizymes was remarkably stable, with ~80% of the initial activity still remaining after 10 reaction cycles. In contrast, the activity of the agarose-immobilised G6PDH was already reduced by 60% after five cycles, although this may in part reflect the loss of agarose-immobilised enzyme beads suspended in the supernatant. Longer separation times and/or additional filtration steps would be required for the optimal recovery of the suspended particles. Forizymes are therefore easier to reuse and recycle, offering the potential to substantially reduce production costs and energy consumption during biocatalytic processes^1^.

### Assembly of forizymes tagged with multiple enzymes

**Figure 4:**
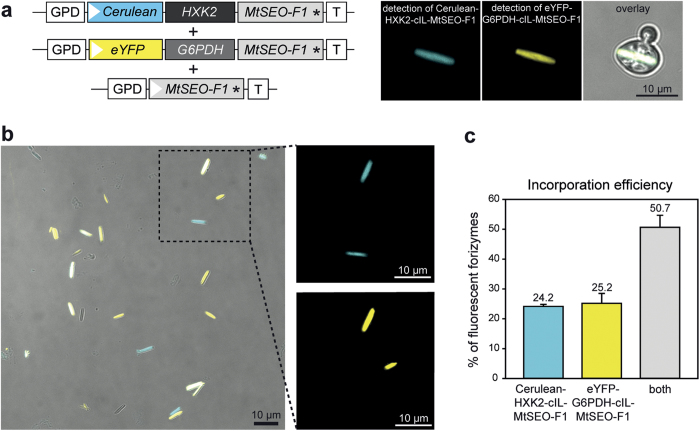
Assembly and incorporation efficiency of multi-enzyme forizymes that display fluorescence-tagged G6PDH and HXK2. (**a**) Vector constructs for the expression of forizymes with two fluorophore-tagged enzymes, and confocal microscopy images of a corresponding yeast cell expressing a Cerulean-HXK2-cIL-MtSEO-F1/eYFP-G6PDH-cIL-MtSEO-F1/MtSEO-F1 forizyme. GPD: gluceraldehyde-3-phosphate dehydrogenase promoter; white arrowhead indicates start codon; black asterisk indicates stop codon; T indicates terminator. (**b**) Confocal microscopy images of purified forizymes composed of Cerulean-HXK2-cIL-MtSEO-F1/eYFP-G6PDH-cIL-MtSEO-F1/MtSEO-F1. Forizymes contain either both (white in overlay) or only one of the fluorophore-tagged enzyme-MtSEO-F1 fusions (blue or yellow, respectively). (**c**) Percentage of forizymes composed of Cerulean-HXK2-cIL-MtSEO-F1/MtSEO-F1, eYFP-G6PDH-cIL-MtSEO-F1/MtSEO-F1, or both fluorophore-enzyme fusions. Data are shown as means ± SD of two independent purifications with n = 183 fluorescent forizymes counted.

Many catalytic processes in lab-on-a-chip devices or biosensors rely on reaction cascades involving multiple enzymes. We therefore investigated whether it is possible to immobilise multiple enzymes on the same forizyme body. To address this question, we chose a two-step reaction involving HXK2 and G6PDH as a model system because each enzyme has been shown to function correctly in the context of a monofunctional forizyme. The two enzymes were fused to different fluorescent proteins, and yeast cells co-expressing the three proteins Cerulean-HXK2-cIL-MtSEO-F1, eYFP-G6PDH-cIL-MtSEO-F1 and MtSEO-F1 produced artificial forisomes that emitted Cerulean-derived blue fluorescence as well as eYFP-derived yellow fluorescence confirming that both enzymes had assembled in the same forizyme (Fig. 4a). The unique assembly mechanism of forisomes therefore allows the co-immobilisation of multiple enzymes. We also observed forizymes emitting only one kind of fluorescence, indicating their specific inclusion of HXK2 or G6PDH alone (Fig. 4b). By counting the forizymes emitting blue, yellow or blue + yellow fluorescence in two independent purification batches, we estimated that ~50% of the forizymes contained all three subunits, ~25% were HXK2 forizymes and ~25% were G6PDH forizymes (Fig. 4c). It is possible that the monofunctional forizymes represent yeast lines that had lost one of the extrachromosomal expression vectors or produce one of the fusion proteins in amounts too low to detect. The proportion of bifunctional forizymes could therefore be improved in the future by using a single vector or by the stable integration of the forizyme genes into the yeast genome^41^.

### Forizymes as catalytically active multi-enzyme complexes

**Figure 5:**
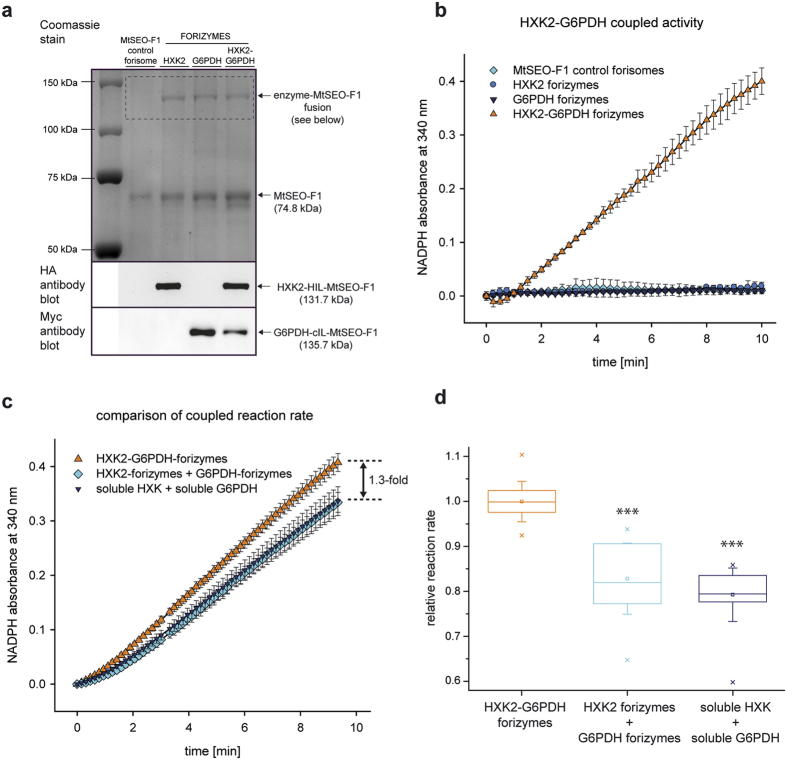
Production and analysis of HXK2-G6PDH forizymes for a two-step reaction cascade. (**a**) SDS-PAGE analysis and immunoblot detection for the verification of HXK2-G6PDH forizyme production. HXK2-G6PDH forizymes are composed of MtSEO-F1 subunits and two enzyme-MtSEO-F1 fusion proteins, namely HXK2-HIL-MtSEO-F1 (HA epitope) and G6PDH-cIL-MtSEO-F1 (Myc epitope). Artificial forisomes composed of only MtSEO-F1, and single enzyme forizymes (HXK2 and G6PDH forizymes) were produced and purified simultaneously as controls. (**b**) Enzyme assay to detect the coupled activity of HXK2-G6PDH forizymes and controls. Coupled enzyme activity was detected using glucose (the HXK2 substrate) in the reaction mixture and no soluble G6PDH was added. Values are shown as means ± SD of technical triplicates from one of three independent forizyme purifications. The initial absorption of each forizyme preparation was set to zero. (**c**) Comparison of the coupled HXK2-G6PDH reaction in HXK2-G6PDH forizymes, mixed monofunctional forizymes and soluble enzymes. The activities of the HXK2 forizymes and soluble HXK were adjusted to be equivalent to the HXK2 activity of the HXK2-G6PDH forizymes, and the same applied to G6PDH activities. The graph shows one representative measurement from three experiments with independent forizyme purifications. Absorption values are means ± SD of triplicate measurements. The initial absorption of each sample was set to zero. (**d**) Relative reaction rate of the coupled HXK2-G6PDH reaction calculated from maximum initial rate velocities obtained in (**c**). One-way ANOVA and a post hoc Holm-Sidak multiple comparison test were carried out to detect significant differences between the coupled reactions of the HXK2-G6PDH forizymes, monofunctional forizymes and soluble enzymes. ***Indicates significant difference (p ≤ 0.001) compared to HXK2-G6PDH forizymes. Boxplots show the 25–75% range (box), median (horizontal line), mean (small rectangle within the box), SD (error bars) and minimum and maximum values (asterisks) from three experiments with independent forizyme purifications.

The catalytic potential of the bifunctional forizymes was investigated in more detail by preparing the construct HXK2-HAL-MtSEO-F1/G6PDH-cIL-MtSEO-F1/MtSEO-F1 without fluorescent tags to produce HXK2-G6PDH forizymes. MtSEO-F1 forisomes and forizymes that lacked either the HXK2-HAL-MtSEO-F1 protein or the G6PDH-cIL-MtSEO-F1 protein were produced as controls, and the correct composition of all the forizymes was confirmed by SDS-PAGE and immunoblot analysis (Fig. 5a). Interestingly, the HXK2-G6PDH forizymes contained a higher portion of enzyme-functionalised subunits and thus a better enzyme-to-carrier ratio than the monofunctional forizymes (Table 2a,b). More precisely, the catalytic mass of the HXK2-G6PDH forizymes was approximately twice that of the monofunctional forizymes (11% *vs* 7% and 5%) suggesting that the presence of two different enzymes allows the assembly of bifunctional forizymes in which the number of catalytic subunits is approximately additive compared to the monofunctional forizymes. Indeed, given that the bifunctional forizymes are presented as a heterogeneous mixture in which half the particles are monofunctional (and thus presumably have the same structure as the monofunctional forizymes produced by expressing only a single enzyme-functionalised subunit; Fig. 4c), then the bifunctional forizymes must have an even higher percentage of catalytic subunits (Table 2b, values in parentheses). These data suggest that, at least in this specific case study, the different functionalised subunits do not interfere with each other’s assembly in the context of the forisome, to the extent that they can be regarded as structurally independent and therefore each independently can assemble with the same density as found in the monofunctional forizymes.

To investigate the catalytic potential of the HXK2-G6PDH forizymes, we first determined the specific activities of the individual enzymes using the corresponding assays, and found that both enzymes remained active when immobilised on the same forizyme and their specific activities were comparable to the equivalent monofunctional forizymes (Table 3). This supports our hypothesis that the enzyme-functionalised subunits effectively behave independently and can assemble and function in the same manner as they do in the monofunctional forizymes. We then tested the sequential activity of the HXK2-G6PDH forizymes using a coupled HXK2-G6PDH enzyme assay, i.e. the assay was fed with glucose (the HXK2 substrate) and we measured NADPH (produced by G6PDH). We duly observed an increase in NADPH absorbance, confirming that the activities of both enzymes were coupled (Fig. 5b).

Multi-enzyme immobilisation systems can be highly efficient because mass transfer limitations are restricted and the close proximity of the enzymes allows the rapid transfer of intermediates by metabolic channelling^13^. To determine whether HXK2-G6PDH forizymes benefit from such effects, the sequential reaction assay was repeated but this time we compared the bifunctional forizymes to equimolar mixtures of the monofunctional forizymes. We found that the specific coupled activity of the monofunctional forizymes was slightly lower than that of the bifunctional forizymes (Table 3). However, the specific activities of each of the single enzymes also differed slightly, which could explain the differences in coupled activity (Table 3). To simplify the comparison, we therefore calculated the reaction efficiency of both systems, which is defined as the quotient of the sequential activity of both enzymes and the activity of HXK2, the first enzyme in the cascade^42^. The bifunctional forizymes showed an efficiency of 43%, i.e. they were more efficient than the coupled monofunctional forizymes (Table 3). This indicates that a higher fraction of glucose-6-phosphate produced by HXK2 was converted when the enzymes were in close proximity, probably because there was limited diffusion from the reaction site thus maintaining a high local concentration of the intermediate^13^,^16^. These values are in good agreement with reaction efficiencies reported in previous studies using other co-immobilised enzymes^42^. Reaction efficiencies close to 100% are unlikely due to the diffusion limitations of both enzymes, but a further increase in efficiency might be achieved by reducing substrate concentrations^43^.

Next, we directly compared the reaction rates of both systems as well as the corresponding soluble enzymes in a coupled enzyme assay using equivalent catalytic units of the individual enzymes. As shown in Fig. 5c,d, the reaction rate of the coupled reaction between HXK2 and G6PDH was significantly (1.3-fold) higher for the HXK2-G6PDH forizymes than the corresponding mixture of monofunctional forizymes or soluble enzymes (p ≤ 0.001, one-way ANOVA and post hoc Holm-Sidak multiple comparison test). Such a difference between soluble and co-immobilised HXK and G6PDH has been observed previously with other carriers (Sepharose and cross-linked acrylamide-acrylic acid copolymers) and was caused by the immediate conversion of glucose-6-phosphate due to the close proximity of the two enzymes^44^. The higher reaction rate and efficiency of the HXK2-G6PDH forizymes is similarly likely to reflect the substrate channelling made possible by immobilising the two enzymes in close proximity. To gain further insight into the reaction kinetics of the monofunctional and bifunctional forizymes, we monitored their initial reaction rates at varying substrate concentrations. Assays were performed using 0.004–0.25 mM glucose-6-phosphate to determine the kinetics for G6PDH, and 0.01–100 mM glucose to determine the kinetics for HXK2 and the HXK2-G6PDH coupled reaction. The initial rates of NADPH formation followed Michaelis-Menten kinetics as a function of substrate concentrations from which *K*_m_ values were derived (Table 4). The *K*_m_ value of HXK2 for glucose was similar in both the monofunctional HXK2 forizymes and the bifunctional HXK2-G6PDH forizymes, suggesting that the HXK2 enzymes fold and function independently of the forizyme background. However, the *K*_m_ of G6PDH for glucose-6-phosphate was higher in the bifunctional forizymes than the monofunctional G6PDH forizymes, which may reflect a difference in G6PDH folding. Nevertheless, there was no significant difference between the *K*_m_ values of the sequential reaction of both enzymes from HXK2-G6PDH forizymes or an equivalent mixture of monofunctional forizymes, indicating that effects such as substrate channelling may compensate for the higher *K*_m_ of G6PDH observed in bifunctional forizymes.

Our results suggest that the co-immobilisation of enzymes on a single forizyme can be used to develop artificial multi-enzyme complexes in which substrate channelling substantially increases the reaction rates. Such forizyme metabolons could be applied in multi-enzyme biosynthesis reactions and for the complex reaction cascades required in cell-free synthetic pathway biotransformation processes^16^. In comparison to other non-ordered co-immobilisation techniques such as cross-linked enzyme aggregates, random co-immobilisation on solid supports and sol-gel encapsulations, where purified enzymes can be mixed in different ratios^45^,^46^, forizymes will require the fine-tuning of enzyme ratios by changing the promoter strength or copy number of the corresponding expression cassettes^47^.

We have combined the stimulus-response characteristics of forisomes and the catalytic activity of enzymes, which may allow the development of single forizymes that act both as a biosensor/valve and as a catalyst in microfluidic devices (Supplementary Fig. S3). For example, our model HXK2 and G6PDH forizymes could be developed into lab-on-a-chip devices for the detection of glucose or ATP, because such devices require micromolecular pump and valve systems as well as enzymatic cascades for the measurement of glucose/ATP concentrations^48^,^49^. The construction of such devices remains a challenging task that will need to be addressed in the future, but our novel forizymes combine biosensor and catalytic properties that would be highly advantageous when integrated into such devices.

## Materials and Methods

### Cloning and expression of eYFP fusion proteins in *S. cerevisiae*

The *S. cerevisiae* Advanced Gateway Destination Vector^50^ pAG424GPD-eYFP-ccdB was used to express N-terminal eYFP fusion proteins of MtSEO-F1 and MtSEO-F4 as previously described^24^. The MtSEO-F1-eYFP C-terminal fusion protein was generated by LR recombination between pAG424GPD-ccdB-eYFP and the previously-described vector pENTR4^TM^-MtSEO-F1*^27^ lacking a translational stop codon, whereas the corresponding MtSEO-F4-eYFP C-terminal fusion was produced in the same manner but using reverse primer 5′-AGA CTC GAG ACA CCA AGA TTG TTT GGT TC-3′ to generate pENTR4^TM^-MtSEO-F4*.

As a control, pAG424GPD-eYFP-ccdB was introduced into *S. cerevisiae* strain INV*Sc*1 (Invitrogen) alone and in combination with each pAG425GPD-MtSEO-F vector^24^ using the frozen yeast method^51^. Transformants were selected at 30 °C on minimal synthetic defined (SD) medium lacking tryptophan or leucine and tryptophan. Likewise, yeast cells were transformed with both pAG424GPD-eYFP-MtSEO-F and pAG424GPD-MtSEO-F-eYFP vectors alone and in combination with each pAG425GPD-MtSEO-F vector.

### Cloning the enzyme-forisome fusion constructs

For the G6PDH-MtSEO-F fusions, synthetic linker sequences (sense 5′-CAT GAG AGA GCT CAA CAT GTT AGC GGC CGC AGA ACA AAA ATT GAT TTC TGA AGA AGA TTT GCC TAG ACC ACC TAC TCC AGC TAA ACA AGA TGC TTA TTG GGC CAT G-3′, antisense 5′-GCC CAA TAA GCA TCT TGT TTA GCT GGA GTA GGT GGT CTA GGC AAA TCT TCT TCA GAA ATC AAT TTT TGT TCT GCG GCC GCT AAC ATG TTG AGC TCT CTC-3′) were annealed and inserted into the NcoI-site of pENTR^TM^-MtSEO-F1 24 and pENTR^TM^-MtSEO-F4 24 resulting in pENTR^TM^-cIL-MtSEO-F1 and pENTR^TM^-cIL-MtSEO-F4. The linker introduced SacI/NotI sites for the insertion of enzyme sequences, a Myc epitope enabling immunodetection, and an IgA protease site for the proteolytic cleavage of the encoded fusion protein. Total RNA was extracted from *S. cerevisiae* cells and used for cDNA synthesis with SuperScript^®^ II Reverse Transcriptase. The G6PDH coding sequence was then amplified using forward primer 5′-AGA GAG CTC ATG AGT GAA GGC CCC G-3′ and reverse primer 5′-AAA GCG GCC GCA TTA TCC TTC GTA TCT TCT G-3′. The product was inserted using SacI/NotI into pENTR^TM^-cIL-MtSEO-F1 and pENTR^TM^-cIL-MtSEO-F4 and the resulting G6PDH-cIL-MtSEO-F fusion sequences were introduced into yeast expression vectors by recombining the pENTR^TM^ vectors with pAG424GPD-ccdB and pAG424GPD-eYFP-ccdB^50^.

The linker construct for the HXK2-MtSEO-F fusions was generated by amplifying pENTR^TM^-cIL-MtSEO-F1 using forward primer 5′-AGA GCG GCC GCA TAC CCA TAC GAT GTT CCT GAC TAT GCG CCT AGA CCA CCT ACT CC-3′ and reverse primer 5′-GGG GTG GTT ATC ATC TGA CAA G-3′, thus replacing the coding sequence of the Myc epitope with the HA epitope. Both the product and pENTR^TM^-cIL-MtSEO-F1 were digested with NotI/NcoI, and ligation resulted in pENTR^TM^-HAL-MtSEO-F1. The HXK2 coding sequence was obtained as described above for G6PDH, using forward primer 5′-AGA GAG CTC ATG GTT CAT TTA GGT CCA A-3′ and reverse primer 5′-AAA GCG GCC GCA GCA CCG ATG ATA CCA AC-3′, followed by digestion with SacI/NotI and insertion into pENTR^TM^-HAL-MtSEO-F1 and pENTR^TM^-cIL-MtSEO-F1, which were subsequently recombined with pAG423GPD-ccdB and pAG423GPD-Cerulean-ccdB^50^ respectively to yield the final HXK2-MtSEO-F expression vectors. All constructs were verified by sequencing.

### Expression of forizymes

To express the eYFP-tagged or untagged G6PDH-cIL-MtSEO-F fusions, the corresponding yeast expression vectors were introduced into strain INV*Sc*1, either with pAG425GPD-MtSEO-F1 for the co-expression of MtSEO-F1^24^ and/or pAG425GPD-MtSEO-F4 for the co-expression of MtSEO-F4^24^. The vector pAG424GPD-eYFP-G6PDH-cIL-MtSEO-F4 was also introduced into the wild-type strain. As an empty vector control for background enzyme activity, cells were co-transformed with pAG424GPD-ccdB^50^ and pAG425GPD-MtSEO-F1. Transformants were selected as described above. To produce HXK2-forizymes, the HXK2-HAL-MtSEO-F1 expression vector was introduced into INV*Sc*1 along with pAG425GPD-MtSEO-F1. A corresponding control was generated by co-transformation with pAG425GPD-MtSEO-F1 and the empty pAG423GPD-ccdB^50^ vector and transformed cells were selected on SD medium lacking leucine and histidine.

As a control for multi-enzyme-tagged forizymes, pAG425GPD-MtSEO-F1 was introduced into Inv*Sc*1 along with pAG423GPD-ccdB and pAG424GPD-ccdB. Further controls were obtained by introducing the same set of vectors, but exchanging each empty expression vector with the corresponding HXK2-HAL-MtSEO-F1 or G6PDH-cIL-MtSEO-F1 vector, as required. Finally, yeast cells were transformed with all three forisome expression vectors, namely pAG425GPD-MtSEO-F1, pAG423GPD-HXK2-HAL-MtSEO-F1 and pAG424GPD-G6PDH-cIL-MtSEO-F1. Likewise, Inv*Sc*1 was transformed with pAG425GPD-MtSEO-F1 and the appropriate eYFP-G6PDH and Cerulean-HXK2 forisome expression vectors to obtain transformants expressing fluorophore-tagged forizymes. Transformants were selected on SD medium lacking leucine, tryptophan and histidine.

### Confocal laser scanning microscopy

Bright field images and fluorescence images of the eYFP fusion proteins expressed in yeast cells were captured using a Leica TCS SP5 confocal microscope as previously described^24^. For the simultaneous detection of eYFP and Cerulean fluorescence, a sequential scan between frames was performed first with excitation/emission wavelengths of 405/457–492 nm to detect Cerulean fluorescence and then with excitation/emission wavelengths of 514/540–580 nm to detect eYFP fluorescence.

Yeast cells expressing fluorescent artificial forisomes were analysed after growth to an optical density (OD) of 1.5 in 3-ml liquid cultures, and were pelleted and resuspended in 100 μl Ca^2+^-free buffer containing 10 mM Tris/HCl (pH 7.2), 10 mM EDTA and 100 mM KCl prior to microscopy. At least three colonies (n = 278–574 yeast cells) were analysed for each eYFP construct and at least three images were obtained for each colony. For eYFP-G6PDH-constructs, four colonies (n = 833–1297 yeast cells) were analysed and five images were obtained for each colony. The number of fluorescent forisomes, fluorescent inclusion bodies and instances of cytosolic fluorescence in each image was counted and set in relation to the total number of fluorescent yeast cells.

### G6PDH activity assay of MtSEO-F subunit combinations

G6PDH activity was measured in yeast cells transformed with the relevant constructs, grown to an OD of 4 in 5-ml cultures for 20 h at 30 °C and 160 rpm. The forizymes/control forisomes were freed by mechanical cell disruption for 45 min at 30 Hz with 750 μl of glass beads (425–600 μm diameter) in an MM 400 bead mill (Retsch, Germany). The cell debris was pelleted and resuspended in 200 μl 250 mM glycylglycine buffer (pH 7.4). The enzyme activity was determined using 10 μl of the cell mixture as described below. We analysed 12 yeast colonies representing each construct to compare the different G6PDH-cIL-MtSEO-F/MtSEO-F combinations.

### Production and purification of forizymes

All forizymes were produced in 200-ml yeast cultures and were purified as previously described^24^ with the following modifications. Briefly, yeast cells were grown to an OD of 5 and digested with 300 U of *Arthrobacter luteus* lyticase following a washing step in Ca^2+^-free Tris buffer (see above) plus a cOmplete Mini protease inhibitor tablet. After mechanical cell disruption for 12 min as described above, the cells were loaded onto a 72.8–105.2% (w/v) Nycodenz^®^ (Medinor AS, Norway) density gradient (10 ml volume) prepared with a gradient mixer in combination with the Econo Gradient Pump (Bio Rad, Germany) and centrifuged for 2.5 h at 4 °C and 247,103.6 x g in an Optima L-70 K ultracentrifuge (Beckmann Coulter, Germany). The band containing the forizymes was isolated, washed with Ca^2+^-free Tris buffer (see above), and analysed by microscopy to determine the purity. If necessary, purification was improved by loading the isolated forizymes onto a second 72.8–105.2% (w/v) Nycodenz^®^ density gradient (5 ml volume). After centrifugation for 1.5 h at 4 °C and 245,418.9 x g, the forizyme band was isolated and resuspended in 800 μl Ca^2+^-free Tris buffer (see above). The production and purification was carried out three times using independent batches, and all subsequent experiments were performed independently for each batch unless indicated otherwise.

### Molecular characterisation and quantification of forizyme yields

Purified forizymes were counted in a Neubauer improved counting chamber and analysed by SDS-PAGE using a 7% resolving gel as previously described^24^. Triplicate aliquots were used for protein quantification by SDS-PAGE against five bovine serum albumin (BSA) standards ranging from 0.075–0.75 μg (applied as duplicates) for which the linear dynamic range was confirmed beforehand. Protein bands were quantified^24^ and used to calculate: a) the yield after purification; b) the proportion of untagged and enzyme-tagged MtSEO-F1 in a single forizyme; c) the ratio of enzyme to carrier protein (based on the molecular mass of the proteins) with the mass of carrier protein calculated as the sum of untagged MtSEO-F1 and MtSEO-F1 in the fusion protein; d) the catalytic mass of the total forizyme mass; and e) the enzyme activity per unit of forizyme protein mass. The separated proteins were also used for western blotting as previously described^27^ using anti-Myc and anti-HA antibodies (Sigma-Aldrich, Germany).

### Enzyme activity assays

The reaction mixture for the detection of G6PDH activity contained 50 mM glycylglycine buffer, 2 mM D-glucose-6-phosphate, 0.67 mM β-NADP and 10 mM magnesium chloride (pH 7.4). The reaction mixture for the detection of HXK2 activity contained 50 mM Tris/HCl buffer, 10 mM magnesium chloride, 100 mM D-glucose, 0.55 mM ATP, 0.34 mM β-NADP (pH 7.6) and 0.2 U soluble *S. cerevisiae* G6PDH (Sigma-Aldrich). Coupled HXK2-G6PDH enzyme activity was detected using the latter reaction mixture without the soluble G6PDH. Unless indicated otherwise, 0.25 μg of forizyme protein was added to each reaction. Triplicate reactions were initiated by adding the substrate and incubated at 30 °C unless indicated otherwise. Assays were performed in microtiter plates with a 300 μl reaction volume and a corresponding path length of 0.794 cm.

Enzyme activity was detected by measuring the formation of NADPH, which was monitored by measuring the absorbance at 340 nm with an Infinite M200 microplate reader (Tecan, Switzerland). Specific enzyme activity was calculated using the Beer-Lambert law and the maximum initial rate of change in absorbance determined using Magellan software (Tecan) and the molar extinction coefficient of NADPH at 340 nm (6.22 mM^−1^ cm^−1^). Results were corrected by subtracting the background activity of blank measurements and/or appropriate control forisomes. One unit was defined as the amount required to oxidise 1.0 μ mole of substrate per minute at 30 °C and pH 7.4 (G6PDH) or pH 7.6 (HXK2/coupled HXK2-G6PDH activity).

For kinetic studies, Michaelis-Menten constants (*K*_m_) were determined from the initial rate measurements of bifunctional HXK2-G6PDH forizymes or an equivalent mixture of HXK2 forizymes and G6PDH forizymes at varying substrate concentrations. The enzyme concentrations were 5 nM G6PDH and 2.5 nM HXK2. The data were fitted to the standard Michaelis-Menten equation and used to estimate *K*_m_ by non-linear regression analysis.

### Stimulus-response characteristics of forizymes

To test the influence of Ca^2+^ on forizyme activity, the reaction mixture was supplemented with 0, 0.1, 1, 7.5 or 10 mM calcium chloride. Forizyme reactivity was monitored with a Leica DMLSF microscope by slowly adding each reaction mixture containing different concentrations of Ca^2+^ until a conformational change was observed. Ca^2+^ was removed by the addition of Ca^2+^ -free Tris buffer containing EDTA (see above).

### Thermal stability of forizymes

To test for thermal stability, purified forizymes and soluble *S. cerevisiae* G6PDH were incubated at 40 °C for 5 h and aliquots were removed after 30, 60, 90, 120, 180, 240 and 300 min of incubation to determine the residual enzyme activity as above.

### Reusability of forizymes

To test for reusability, 0.01 U of purified G6PDH-cIL-MtSEO-F1/MtSEO-F1 forizymes were centrifuged to the bottom of a microtiter plate (10 min at 4 °C and 4,500 x g) containing 100 μl phosphate-buffered saline (PBS) and the non-adsorbed forizymes were evenly distributed on the well bottom by pipetting up and down, followed by centrifugation as above. This step was repeated and the supernatant was discarded. Likewise, 0.01 U of agarose-immobilised *S. cerevisiae* G6PDH (Sigma-Aldrich) was centrifuged to the well bottom after washing in 250 mM glycylglycine buffer (pH 7.4). Adsorption of the forizymes was verified using a Leica DMLSF microscope, by cutting out a piece of the reference well bottom with a scalpel. The enzyme assay was initiated by adding the assay solution followed by the substrate glucose-6-phosphate. After 15 min, the reaction was stopped by removing the assay solution and a new reaction cycle was initiated by the addition of fresh reaction buffer including the substrate. This step was repeated for 10 assay cycles.

### Comparison of coupled reaction efficiency

Coupled reactions were compared using: a) the same mass of forizymes, i.e. 0.25 μg HXK2-G6PDH forizymes and mixture of 0.25 μg HXK2 forizymes plus 0.25 μg G6PDH forizymes; or b) the same activity (total units per assay) of HXK from HXK2-G6PDH forizymes, HXK2 forizymes and soluble HXK, and of G6PDH from HXK2-G6PDH forizymes, G6PDH forizymes and soluble G6PDH. Equal activities were confirmed in corresponding HXK2 and G6PDH assays followed by one-way ANOVA. Soluble *S. cerevisiae* HXK and G6PDH were obtained from Sigma-Aldrich. The assays were carried out as six technical replicates.

### Statistical analysis

One-way ANOVA was applied to mean enzyme activities to detect differences between normally distributed datasets. Any datasets that failed the Shapiro-Wilk normality test were compared using Kruskal-Wallis one-way analysis on ranks. As indicated, post hoc Holm Sidak or Tukey pairwise multiple comparison tests were carried out to isolate groups that showed significant differences. An alpha level of 0.01 was applied to all tests.

## Additional Information

**How to cite this article**: Visser, F. *et al*. Forizymes – functionalised artificial forisomes as a platform for the production and immobilisation of single enzymes and multi-enzyme complexes. *Sci. Rep*. **6**, 30839; doi: 10.1038/srep30839 (2016).

## Supplementary Material

Supplementary Information

## Figures and Tables

**Table 1:** Proportion of yeast cells expressing eYFP-tagged MtSEO-F1 or MtSEO-F4 proteins that form fluorescent forisomes.

	Expressed protein (s)	Number of cells with forisomes per 100 fluorescent yeast cells
a	eYFP-MtSEO-F4	67 ± 23
	eYFP-MtSEO-F1/MtSEO-F4	35 ± 5
	eYFP-MtSEO-F1/MtSEO-F1	84 ± 9
	eYFP-MtSEO-F4/MtSEO-F1	75 ± 5
	eYFP-MtSEO-F4/MtSEO-F4	94 ± 2
	MtSEO-F1-eYFP/MtSEO-F1	21 ± 11
	MtSEO-F1-eYFP/MtSEO-F4	25 ± 5
	MtSEO-F4-eYFP/MtSEO-F1	40 ± 11
	MtSEO-F4-eYFP/MtSEO-F4	70 ± 15
b	eYFP-G6PDH-cIL-MtSEO-F4	0 ± 0
	eYFP-G6PDH-cIL-MtSEO-F1/MtSEO-F1	61 ± 9
	eYFP-G6PDH-cIL-MtSEO-F4/MtSEO-F1	42 ± 4
	eYFP-G6PDH-cIL-MtSEO-F4/MtSEO-F4	49 ± 13

(**a**) Analysis of robustness (tendency to form forisomes) of eYFP-MtSEO-F subunit combinations in order to identify the most appropriate MtSEO-F1/MtSEO-F4 combination and translational fusion site for the eYFP tag. (**b**) Robustness of eYFP-enzyme-fused MtSEO-F subunit combinations. The table shows mean numbers ± SD of yeast cells with properly assembled, fluorescent forisomes counted per 100 fluorescent yeast cells. Three (**a**) or four (**b**) yeast colonies were analysed for each construct.

**Table 2:** Protein composition and the catalytic proportion of forizymes.

		Protein composition [%]		Loading capacity	
		Enzyme-MtSEO-F1 fusion	MtSEO-F1	Enzyme:carrier ratio^1^	Catalytic mass [%]
a	G6PDH forizyme	18.6 ± 4.1	81.4 ± 4.1	1 : 15.0 ± 3.9	7.0 ± 1.8
	HXK2 forizyme	12.7 ± 1.8	87.3 ± 1.8	1 : 20.6 ± 0.8	4.9 ± 0.2
b	HXK2-G6PDH forizyme	27.9 ± 2.5 (39.7)^2^	72.1 ± 2.5 (60.3)	1 : 9.5 ± 1.0 (6.6)^3^	10.6 ± 1.2 (15.1)^3^

^1^Carrier = total non-catalytic mass (untagged MtSEO-F1 + MtSEO-F1 in the enzyme-MtSEO-F1 fusion protein).

^2^Calculated as the sum of HXK2-HAL-MtSEO-F1 and G6PDH-cIL-MtSEO-F1.

^3^Enzyme proportion calculated as the sum of HXK2 and G6PDH.

(**a**) Analysis of monofunctional forizymes displaying G6PDH or HXK2. (**b**) Analysis of bifunctional forizymes displaying the two enzymes HXK2 and G6PDH. Values were based on quantitative SDS-PAGE and assume that all forizymes (of one type) are based on approximately the same proportions of enzyme-functionalised and native subunits. Because the distribution is different in HXK2-G6PDH forizymes (Fig. 4b,c) the values in parentheses in **b** estimate the proportion of HXK2-G6PDH forizymes that carry both enzymes simultaneously. Values are means ± SD of three independent forizyme purifications.

**Table 3:** Comparison of catalytic parameters of monofunctional and bifunctional forizymes.

	Specific activity [U/g forizyme]			Efficiency of coupled reaction [%]
	HXK2	G6PDH	Coupled reaction	
**G6PDH forizymes**	—	10,800 ± 3,300	8,000 ± 1,400	31.1 ± 6.4
**HXK2 forizymes**	25,700 ± 2,900	—		
**HXK2-G6PDH forizymes**	23,500 ± 7,600	13,800 ± 3,200	9,400 ± 3,300	42.7 ± 7.6

Data are shown as means ± SD from three independent forizyme purifications. Specific activities were corrected for the appropriate control forisomes. Efficiency was calculated as the quotient of coupled activity and the activity of HXK2, the first enzyme in the cascade.

**Table 4:** Michaelis-Menten constants of monofunctional and bifunctional forizymes.

Enzyme	Substrate	Forizyme	*K*_m_ [mM]
HXK2	glucose	monofunctional	0.316 ± 0.052^ns^
		bifunctional	0.461 ± 0.115^ns^
G6PDH	glucose-6-phosphate	monofunctional	0.045 ± 0.010^a^
		bifunctional	0.064 ± 0.005^a^
HXK2-G6PDH	glucose	monofunctional	0.275 ± 0.058^ns^
		bifunctional	0.310 ± 0.058^ns^

*K*_m_ values are shown as means ± SD from three experiments. Values in each row were compared by two-tailed t-test (for HXK2 and G6PDH) or Mann-Whitney rank sum test (for HXK2-G6PDH). ^ns^no significant difference (p = 0.094 for HXK2, p = 0.393 for HXK2-G6PDH). ^a^significant difference between the two *K*_m_ values (p = 0.015).
