# Supplementary material for: Forizymes – functionalised artificial forisomes as a platform for the production and immobilisation of single enzymes and multi-enzyme complexes
Source: Sci Rep. 2016 Aug 9;6:30839. doi: 10.1038/srep30839 (PMC4977538; doi:10.1038/srep30839)
Supplement: Supplementary Information [file srep30839-s1.pdf]

Supplementary Information

Forizymes – functionalised artificial forisomes as a platform for the production and immobilisation of single enzymes and multi-enzyme complexes

Franziska Visser[1,+], Boje Müller[1,+], Judith Rose[2], Dirk Prüfer[1,2*] & Gundula A Noll[1]

[1]University of Münster, Institute of Plant Biology and Biotechnology, Münster, 48143, Germany.

[2]Fraunhofer Institute for Molecular Biology and Applied Ecology IME, Münster, 48143, Germany.

*corresponding author: dpruefer@uni-muenster.de

[+]these authors contributed equally to this work

Number of supplemental figures: 3

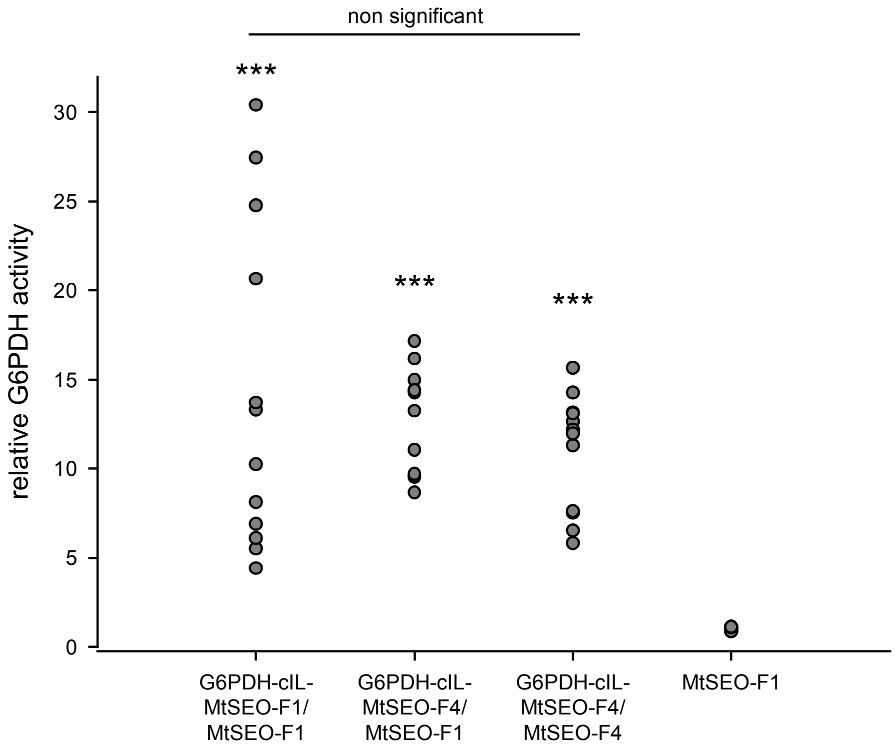

Supplementary Figure S1: Catalytic activity of different G6PDH-MtSEO-F subunit combinations. G6PDH activity was measured in yeast cell extracts expressing the candidate combinations. Values show the activity of single yeast colonies (n = 12) calculated in relation to the activity of control cells expressing forisomes without G6PDH (MtSEO-F1). Significant differences between the combinations were detected with Kruskal-Wallis one-way analysis of variance on ranks and post hoc Tukey test with an alpha level of 0.01. *** indicates significant difference ($p < 0.001$) compared to MtSEO-F1.

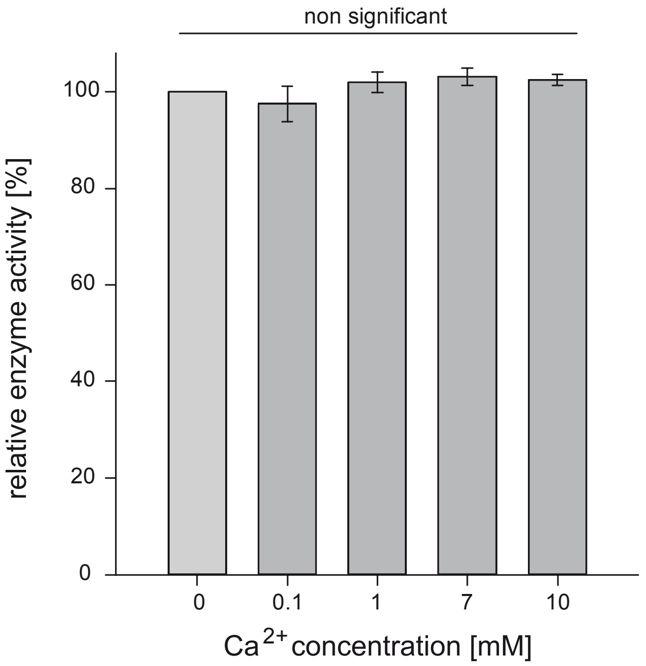

Supplementary Figure S2: G6PDH forizyme activity in response to rising Ca^{2+} concentrations. Effect of Ca^{2+}-induced conformational change on the catalytic activity of G6PDH forizymes. Relative enzyme activity is shown as a percentage of activity without the addition of Ca^{2+} to the reaction buffer. Data are shown as means ±SD of three independent experiments for each purification. One-way ANOVA with an alpha level of 0.01 detected no significant differences ($p = 0.055$) between activities at the different Ca^{2+} concentrations.

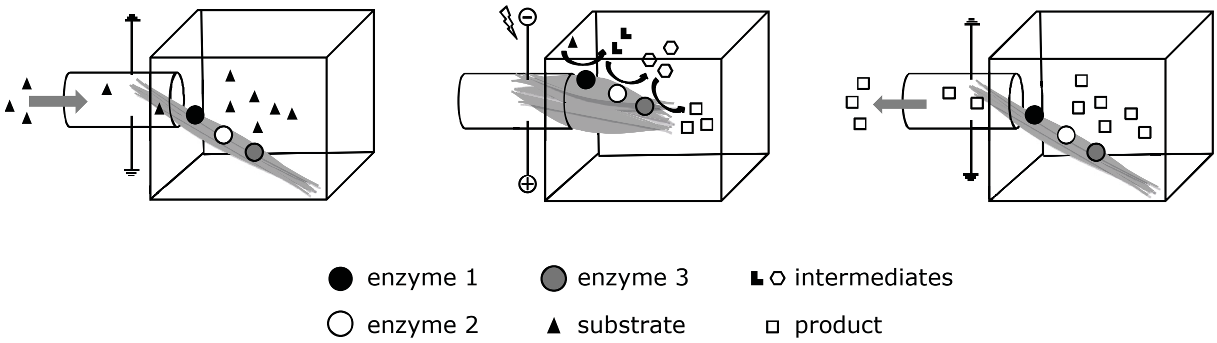

Supplementary Figure S3: Potential application of forizymes. Multi-enzyme forizymes could be used as the basis of a microfluidic device in which the forizyme serves both as an electrically regulated valve and as a platform for a multi-step enzymatic cascade within a microreaction chamber. The condensed conformation of the forizyme allows the entry of substrate into the microreaction chamber. Upon the electrically induced conformational change, the forizyme plugs the channel, facilitating the accumulation of the reaction product within the chamber. The product can be released when the forizyme changes back into the original condensed conformation.
